# In Vitro Inhibitory Effects and Molecular Mechanism of Four Theaflavins on Isozymes of CYP450 and UGTs

**DOI:** 10.3390/foods14162822

**Published:** 2025-08-14

**Authors:** Lin Hu, Zhuohan Hu, Junying Peng, Aixiang Hou, Zhubing Hao, Zhongqin Wu, Yan Li, Ke Li, Zongjun Li, Zhonghua Liu, Yu Xiao, Yuanliang Wang

**Affiliations:** 1 College of Food Science and Technology, Hunan Agricultural University, Changsha 410128, China; hulin455@163.com (L.H.); aixianghou@163.com (A.H.); 17835423748@163.com (Y.L.); leeke14@163.com (K.L.); hnlizongjun@163.com (Z.L.); 2 Hunan Drug Inspection Center, Changsha 410001, China; 3 RILD In Vitro Technologies (Changsha) Co., Ltd., Changsha 410137, China; huzh@rild-biotech.com (Z.H.); pengjy@rild-biotech.com (J.P.); haozb@rild-biotech.com (Z.H.); 4 School of Pharmaceutical and Bioengineering, Hunan Chemical Vocational Technology College, Zhuzhou 412000, China; hnwuzhongqin@163.com; 5 Key Laboratory of Ministry of Education for Tea Science, College of Horticulture, Hunan Agricultural University, Changsha 410128, China

**Keywords:** theaflavins, CYP450, UGT, inhibitory effect, molecular docking analysis

## Abstract

Theaflavins, benzotropolone compounds formed during black tea processing via catechin condensation, have drawn attention for their potential health benefits and diverse biological effects. This study evaluated the inhibitory effects of four theaflavin monomers—theaflavin-3′-gallate, theaflavin-3,3′-digallate, theaflavin-3-gallate, and theaflavin—on eight CYP450 enzymes using pooled human liver microsomes and specific probe substrates, and seven UGT enzymes using human recombinant UGT enzymes and specific probe substrates. Theaflavin-3′-gallate moderately inhibited CYP1A2-catalyzed phenacetin metabolism and CYP2C8-mediated amodiaquine metabolism, with IC_50_ values of 8.67 μM and 10–20 μM, respectively. Theaflavin-3,3′-digallate exhibited similar effects. Both compounds showed negligible inhibition with other CYP enzymes. In UGT assays, theaflavin-3′-gallate and theaflavin-3,3′-digallate moderately inhibited UGT1A1- and UGT1A3-mediated beta-estradiol glucuronidation (IC_50_: 1.40–5.22 μM), with weak or no effects on other UGT enzymes. Molecular docking revealed that CYP1A2-theaflavin-3′-gallate and CYP2C8-theaflavin-3,3′-digallate interactions were non-competitive, primarily mediated by hydrogen bonding and π-interactions. UGT1A1-theaflavin interactions suggested non-competitive inhibition, while UGT1A3-theaflavin interactions indicated competitive inhibition. Other enzyme-theaflavin interactions exhibited minimal binding energy differences, implying mixed-type inhibition. These findings highlight the selective inhibitory effects of theaflavins on specific hepatic enzymes, with potential implications for nutrient interactions, particularly for nutrients metabolized by CYP1A2, CYP2C8, UGT1A1, and UGT1A3. Further research is needed to explore the in vivo relevance and assess the dietary implications of theaflavin-rich black tea in nutrition and metabolism.

## 1. Introduction

Theaflavins are a group of compounds featuring a benzene ring and a diketone structure, created by the condensation of catechins in the presence of polyphenol oxidase during black tea processing [1,2]. Due to their significant theoretical research value and extensive application potential, particularly in food health, the research and development of theaflavin and its gallate have garnered considerable interest in tea research [3,4]. Theaflavins are renowned for their numerous health benefits, including aiding in weight management, regulating blood sugar levels, and preventing lifestyle-related conditions such as obesity, cancer, atherosclerosis, inflammation, infections, osteoporosis, and dental decay [5,6]. These wide-ranging advantages underscore the significance of theaflavins in promoting overall well-being through dietary consumption. As natural constituents of black tea, theaflavins present a safer and potentially more effective option compared to synthetic health supplements [7]. Currently, there is no substantial evidence to suggest that theaflavins are naturally present in significant quantities in other foods. The unique processing steps involved in tea production, specifically the oxidation and fermentation of tea leaves, are crucial for the formation of these compounds. Their natural origin aligns with the growing consumer preference for organic health products. Incorporating theaflavins into functional foods and beverages not only provides nutritional value but also contributes to health improvement and disease prevention [8]. This significantly enhances the appeal of everyday food items to health-conscious consumers. Theaflavin is a potent antioxidant that helps combat oxidative stress and reduce the risk of chronic diseases. Its strong antioxidant properties make it an attractive ingredient for developing foods aimed at improving overall health and longevity [9]. Theaflavin and its gallate (Supplementary Figure S1A–D) meet this demand by offering scientifically backed health benefits.

Cytochrome P450 enzymes (CYP450) are a large family of enzymes that play a critical role in metabolizing various substances, including dietary compounds and endogenous substances [10]. These enzymes are primarily found in the liver but are also present in other tissues. Their involvement in the metabolism of theaflavins is of particular interest [11]. CYP450 is crucial for understanding nutrient interactions, metabolic effectiveness, and diet–drug interactions. Natural compounds, like theaflavins, can also interact with CYP450 enzymes, influencing the metabolism of other dietary components and leading to nutrient interactions. Understanding how theaflavins and similar compounds modulate CYP450 activity is essential for optimizing nutritional therapies and supporting personalized nutrition strategies. Previous studies have explored the impact of polyphenols like flavonoids and catechins on CYP450 enzymes, but the effects of theaflavins remain underexplored [12]. However, recent research indicates that theaflavins exhibit varying inhibitory effects on different CYP450 isozymes, suggesting the need for further investigation into their potential interactions with these enzymes. While previous studies have explored the effects of various polyphenols on CYP450 enzymes, research specifically investigating the inhibitory activity of theaflavins remains scarce. Our study aims to fill this gap by examining theaflavins’ interactions with P450 enzymes, offering new insights into their metabolic effects. Given that theaflavins possess significant biofunctional activity, understanding their inhibitory impact on P450 enzymes is crucial [13]. Research indicates that theaflavins exhibit varying inhibitory effects on different CYP450 isozymes. The modulation of CYP450 enzyme activity by theaflavins has significant implications for dietary planning. Regular consumption of black tea or theaflavin supplements may necessitate adjustments in nutrient intake or monitoring the levels of dietary components, particularly those metabolized by the affected CYP450 enzymes. Understanding the selective inhibition of CYP450 enzymes by theaflavins could be leveraged to modulate nutrient metabolism in a controlled manner, potentially improving health outcomes for certain individuals. Theaflavins from black tea exhibit selective inhibitory effects on specific CYP450 enzymes, notably CYP1A2 and CYP2C8 [13]. These interactions underscore the potential for diet–nutrient interactions, which must be considered in nutritional practice [14].

UDP-glucuronosyltransferases (UGTs) are a family of phase II detoxification enzymes that play a crucial role in the metabolism and elimination of various endogenous and exogenous compounds, including theaflavins found in black tea [15]. These enzymes are primarily located in the liver but are also present in other tissues such as the intestine and kidneys [16]. UGTs are responsible for the detoxification and elimination of many natural substances, including dietary polyphenols and metabolites. The inhibition of UGTs can lead to decreased clearance of these compounds, resulting in prolonged presence in the body and potential metabolic imbalances. Theaflavins play a significant role in modulating the activity of UGTs. UGTs are crucial enzymes in phase II drug metabolism, responsible for the glucuronidation of a wide range of endogenous and exogenous compounds, thereby facilitating their excretion [17]. Although there is substantial research on the inhibitory effects of various natural products on UGTs, specific studies on theaflavins are relatively sparse.

While significant progress has been made in understanding the roles of cytochrome CYP450 and UGTs in the metabolism of theaflavins, many intricacies remain unexplored. Our study employs a rigorous methodological approach involving in vitro enzyme inhibition assays to investigate the inhibitory effects of theaflavins on CYP450 and UGT enzymes. Our research results are significant and valuable, as they enhance our understanding of the metabolic interactions between theaflavins and key enzymes, inform clinical practices, and contribute to public health knowledge. To elucidate the molecular interactions underlying these inhibitory effects, we applied molecular docking techniques, focusing on the binding characteristics between theaflavins and key hepatic CYP and UGT isoforms [18].

The innovation of this study lies in its comprehensive evaluation of the inhibitory effects of theaflavin monomers on a broad range of CYP450 and UGT enzymes, shedding light on their selective interactions. Unlike previous studies, we not only identified theaflavin-3′-gallate and theaflavin-3,3′-digallate as non-competitive inhibitors of CYP1A2 and CYP2C8 but also explored the molecular mechanisms behind these interactions through molecular docking analysis. Our study further demonstrates the potential of specific theaflavin derivatives to selectively inhibit UGT1A1 and UGT1A3, offering insights into the diverse interaction patterns that could influence nutrient metabolism. These findings present a novel perspective on the role of theaflavins in modulating hepatic enzyme activity, with implications for both dietary and metabolic contexts.

## 2. Materials and Methods

### 2.1. Reagents and Instruments

We purchased Nicotinamide adenine dinucleotide phosphate (NADPH) (10041939103) from Roche (F. Hoffmann-La Roche Ltd., Basel, Switzerland), phenacetin (77440), amodiaquine (A2799), diclofenac sodium (D6899), α-naphthoflavone (N5757), tolbutamide (T0891), thiotepa (T6069), uridine diphosphate glucuronic acid (UDPGA) (U6751), β-estradiol (E8875), silybin (S0417), trifluoperazine (T8516), and diclofenac sodium (D6899) were purchased from Sigma (Sigma-Aldrich Corporation, St. Louis, MO, USA); bupropion (B3649), montelukast (M2340), ketoconazole (K0045), acetaminophen (H0190), harmine (H0003) were purchased from TCI (Tokyo Chemical Industry Co., Ltd., Tokyo, Japan); dextromethorphan (75469A) was purchased from Damas-beta (Shanghai, China); S-mephenytoin (M225000), sulfabenzamide (178125), and quinidine (272633) were purchased from J&K (J&K Scientific, Beijing, China); testosterone (DRE-C17322500) was purchased from Dr. Ehrenstorfer (Augsburg, Germany); midazolam (171265) was purchased from the National Institutes for Food and Drug Control of China (Beijing, China); ticlopidine (T872159) was purchased from Macklin(Macklin Biochemical Co., Ltd., Shanghai, China), hydroxybupropion (BUP-16-007) was purchased from Acanthus (Acanthus Pharmaceuticals, San Diego, CA, USA); 4-hydroxy diclofenac (GC42407), 4-hydroxy mephenytoin (GC33522), 1-hydroxy midazolam (GC41995) were purchased from Glpbio (GLPBIO, Inc., Dallas, TX, USA); dextrorphan (BCP24334) was purchased from Biochampatner (Shanghai, China); N-desethyl amodiaquine (ZC-25951) and 6β-hydroxy testosterone (ZC-24060) were purchased from Shanghai ZZBIO (Shanghai ZZBIO Co., Ltd., Shanghai, China); 7-hydroxy-4-(trifluoromethyl) coumarin (368512) was purchased from Aldrich (Sigma-Aldrich Corporation, St. Louis, MO, USA); midostaurin (BD305504) was purchased from Bide Pharmatech (Bide Pharmatech Ltd., Shanghai, China). The automatic sampling system (HTS PAL) was purchased from CTC Analytics AG, located in Basel, Switzerland; the liquid chromatograph (LC 20AD) was purchased from Shimadzu Corporation, located in Kyoto, Japan); the mass spectrometer (API5000) was purchased from Applied Biosystems located in Toronto, ON, Canada.

### 2.2. Inhibition of CYP450 by Theaflavins

To evaluate the potential inhibitory effects of theaflavin (A29IB214177, Shanghai Yuanye Bio-Technology Co., Ltd., Shanghai, China), theaflavin-3′-gallate (S01IB224831, Shanghai Yuanye Bio-Technology Co., Ltd., Shanghai, China), theaflavin-3-gallate (F03IB202755, Shanghai Yuanye Bio-Technology Co., Ltd., Shanghai, China), and theaflavin-3,3′-digallate (S16HB194927, Shanghai Yuanye Bio-Technology Co., Ltd., Shanghai, China) on the activities of major isoenzymes (CYP2D6, CYP1A2, CYP2C8, CYP2B6, CYP2C19, CYP2C9, and CYP3A4), human liver microsomes were obtained from the supplier BIOIVT, which provided information about the human liver microsome donors, including clinical serum test results. The virological tests for AIDS, hepatitis B, and hepatitis C of the donors were negative. The final reaction volume was 150 µL, and the reaction was carried out in a 96-well deep-well cell plate made of polypropylene. The reaction was conducted at a temperature of 37 °C. To ensure that the reaction time was within the linear range of the metabolic rate and to avoid data distortion caused by substrate depletion or product feedback inhibition, the reaction time for each substrate varied as follows: the incubation time for detecting CYP3A4 enzyme activity was 5 min; the incubation time for detecting CYP2B6, CYP2C8, CYP2C9, and CYP2D6 enzyme activities was 10 min each; the incubation time for detecting CYP1A2 and CYP2C19 enzyme activities was 30 min each. The reaction solvent was phosphate-buffered saline with a pH of 7.40 ± 0.02, and the final concentration of all organic solvents in the reaction system, such as methanol, acetonitrile, and dimethyl sulfoxide, did not exceed 1% [19]. The batch of human liver microsomes used in the experiment had been used multiple times in our experimental system and had successfully supported drug clinical submissions. Therefore, internal historical data were deemed more accurate for reference compared to the literature data.

### 2.3. Inhibition of UGTs by Theaflavins

To evaluate the inhibitory effects of theaflavin derivatives (theaflavin-3′-gallate, theaflavin, theaflavin-3-gallate, and theaflavin-3,3′-digallate) on UGT isoenzymes (UGT1A3, UGT1A1, UGT1A9, UGT1A4, UGT1A6, UGT2B7, and UGT2B15), human recombinant UGT enzymes were obtained from Cypex (San Diego, CA, USA) and stored according to the manufacturer’s instructions at −80 °C until use. These enzymes were then incubated with each compound (Table 1). Subsequently, the corresponding substrates for each UGT isoenzyme were added. The inhibitory effects of the respective products were evaluated by measuring their generation. The metabolites of UGT isoenzymes were identified as estradiol-3-glucuronide, estradiol-3-glucuronide, trifluoperazine-glucuronide, 4-trifluoromethylumbelliferone-D-glucuronide, 4-trifluoromethylumbelliferone-D-glucuronide, 4-trifluoromethylumbelliferone-D-glucuronide, and 4-trifluoromethylumbelliferone-D-glucuronide, respectively. Each incubation mixture was prepared in triplicate, containing 0.25 mg/mL recombinant enzyme, 5 mM UDPGA, and 10 mM propylthiouracil, among other conditions.

### 2.4. LC-MS/MS Analysis

The AB SCIEX 4000/5000 HPLC-MS/MS system, integrated with the HTS PAL autosampler (CTC Analytics AG, Basel, Switzerland) and Shimadzu LC 20 series HPLC (Shimadzu Corporation, Kyoto, Japan), was used for analysis. Data acquisition and processing were carried out using Analyst 1.6.3 software. Metabolites of CYP450 and UGT substrates were separated on a Hydro-RP 80Å 30 × 2 mm, 4 μm column (Phenomenex) at 40 °C. The mobile phase consisted of solvent A (0.1% formic acid in water) and solvent B (acetonitrile with 0.1% formic acid), using a linear gradient at a flow rate of 450 μL/min. Multiple reaction monitoring (MRM) was performed to detect protonated ions of analytes in both positive and negative ion modes. Operational parameters included collision, curtain, nebulizer, and auxiliary gases set at 10 psi, 20 psi, 50 psi, and 55 psi, respectively. The ion spray voltage was set to 5500 V for the positive mode and −4500 V for the negative mode. The ion spray temperature was set at 550 °C. The process of metabolite identification was executed through the following steps: Preparation of standards and samples, probe substrates, inhibitors, and known metabolites were prepared at the specified concentrations (as outlined in Table 1 and Table 2) [19]. Samples were created by incubating the substrates with the respective inhibitors in the presence of a matrix. The samples were introduced into the HPLC-MS/MS system for chromatographic separation and detection. Chromatographic separation was then performed as previously described. The mass spectrometer functioned in MRM mode to identify specific transitions of the metabolites. Each metabolite was tracked by its unique multiple reaction monitoring (MRM) transition, which includes the precursor and specific fragment ions. Data acquisition and processing were conducted using Analyst 1.6.3 software. The retention times and MRM transitions of metabolites were compared to authentic standards for identification. Quantification was achieved by calculating the area under the curve (AUC) for each MRM transition. Metabolite identities were confirmed by matching retention times and transitions with those of the standards. Additional validation was conducted by examining the fragmentation patterns of the metabolites and comparing them with known standards.

### 2.5. Molecular Docking Analysis

In this study, we investigated the interactions of the following four theaflavin monomers: theaflavin-3′-gallate, theaflavin-3,3′-digallate, theaflavin-3-gallate, and theaflavin, with key hepatic CYP450 and UGT isoforms using molecular docking techniques [20]. The chemical structure of theaflavin was obtained using its Compound Identifier (CID) from PubChem (https://pubchem.ncbi.nlm.nih.gov/). If a three-dimensional (3D) structure was not available on PubChem, we manually created it using GaussView. Subsequently, ligands were subjected to geometry optimization and energy minimization using Gaussian software 20 (Gaussian, Inc., Pittsburgh, PA, USA) to confirm their structural accuracy prior to docking. This step ensures that the ligands are in their most stable conformation for accurate interaction studies. The 3D structures of the UGT and CYP450 isoforms, including UGT1A1, UGT1A3, UGT1A4, CYP1A2, and CYP2C8, were sourced from the Protein Data Bank (PDB) (Supplementary Table S1). For proteins not available in 3D format on PDB, the amino acid sequences were obtained, and structural modeling was conducted using AlphaFold3 (https://alphafoldserver.com/). To ensure the accuracy of the AlphaFold models, we performed validation using Ramachandran plot analysis and comparison with known structures when available. This ensured that high-quality structures were used for accurate docking results. All protein and ligand files were prepared for docking using AutoDock Tools 1.5.7. The docking grid box was centered over the protein’s active site, with a cubic box size of 20 Å per side to ensure sufficient space for ligand flexibility. In cases of non-competitive inhibition, the box size was expanded to 40 or 60 Å to encompass alternative binding regions. Molecular docking was conducted using AutoDock Vina. The grid spacing was set to 0.375 Å, and a maximum of 100 conformations was allowed for each docking trial. Conformational sampling and scoring were carried out using a genetic algorithm, which enabled efficient exploration of potential binding modes. Docking scores were evaluated, with the optimal docked conformation selected based on binding energy, geometric stability, and alignment with known binding pocket features.

### 2.6. Data Analysis

In accordance with the DDI guidelines from the NMPA and the US FDA, the inhibition of CYP1A2, 2B6, 2C8, 2C9, 2C19, 2D6, and 3A4 activity by the test article was assessed. The inhibitory effect was determined by measuring the reduction in metabolite formation of respective probe substrates, expressed as relative activity compared to the negative control (NC), using the following formula: Relative activity (% of NC) = (formation of metabolites in test or PC/formation in NC) × 100. If the test article exhibits a significant concentration-dependent inhibitory effect on enzyme activity, the half-maximal inhibitory concentration (IC_50_) and its 95% confidence interval are determined using Prism software 10.1 (GraphPad Software, Inc., San Diego, CA, USA). The model formula is as follows: Y = 100/(1 + 10^X−LogIC50^), where X is Log (µM), and Y is relative activity (% of NC). Data processing: When the calculated value is less than 100, three significant digits are retained. When the calculated value is greater than or equal to 100, only the integer part is retained. The rounding errors will not affect the authenticity of the data.

## 3. Results

### 3.1. Inhibition of CYP450 Activities by Theaflavins in HLMs

**Figure 1:**
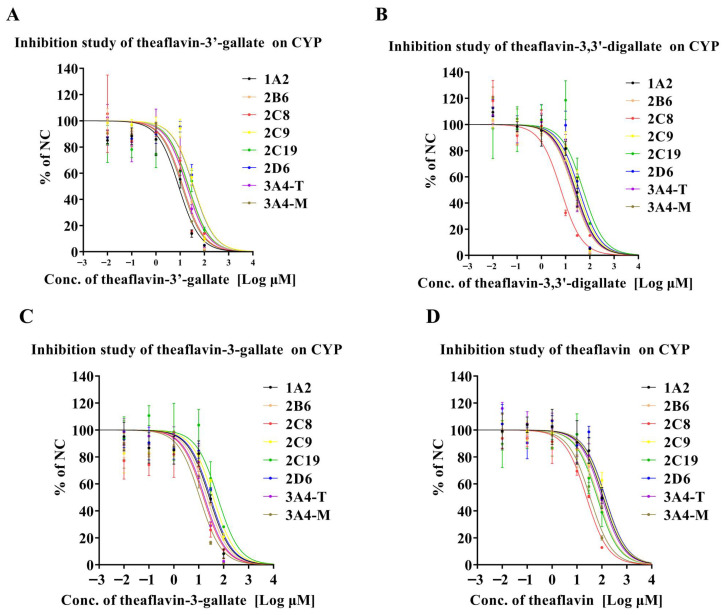
Inhibition of eight CYP enzymes by theaflavin derivatives in pooled HLMs. (**A**) Theaflavin-3′-gallate. (**B**) Theaflavin-3,3′-digallate. (**C**) Theaflavin-3-gallate. (**D**) Theaflavin. Each panel shows the effects on CYP1A2, CYP2B6, CYP2C8, CYP2C9, CYP2C19, CYP2D6, CYP3A4-T, and CYP3A4-M. Specifically, the abbreviations 1A2, 2B6, 2C8, 2C9, 2C19, 2D6, 3A4-T, and 3A4-M refer to CYP1A2, CYP2B6, CYP2C8, CYP2C9, CYP2C19, CYP2D6, CYP3A4-T, and CYP3A4-M, respectively. Results are expressed as mean values ± standard deviation (n = 3).

Figure 1A summarizes the inhibitory effects of theaflavin-3′-gallate on CYP450 enzymes. Theaflavin-3′-gallate moderately inhibited CYP1A2-mediated phenacetin metabolism, with an IC50 value of 8.67 μM. It also exhibited weak inhibition of CYP2C8, CYP3A4-T, and CYP3A4-M, with IC50 values ranging from 10 to 20 μM. However, it showed negligible inhibition on CYP2B6, CYP2C9, CYP2C19, and CYP2D6 activities, with IC50 values exceeding 20 μM. Figure 1B illustrates the inhibition of CYP450 enzymes by theaflavin-3,3′-digallate. Similar to theaflavin-3′-gallate, theaflavin-3,3′-digallate moderately inhibited CYP2C8-mediated amodiaquine metabolism (IC50 = 6.40 μM) but demonstrated minimal inhibition on other CYP450 enzymes, including CYP1A2, CYP2B6, CYP2C9, CYP2C19, CYP2D6, CYP3A4-T, and CYP3A4-M, all with IC50 values exceeding 20 μM. Figure 1C summarizes the effects of theaflavin-3-gallate on CYP450 enzymes. Theaflavin-3-gallate exhibited weak inhibition of CYP2C8, CYP3A4-T, and CYP3A4-M, with IC50 values ranging from 10 to 20 μM, while showing negligible inhibition on other CYP450 enzymes with IC50 values exceeding 20 μM. Figure 1D shows the inhibitory effects of theaflavin on CYP450 enzymes. Theaflavin demonstrated negligible inhibition on all enzymes tested, with IC50 values exceeding 20 μM. Statistical analyses were performed to determine the significance of these differences. Our data indicates that different theaflavin glycoside structures exhibit significant differences in their inhibitory activities on CYP enzymes.

### 3.2. Inhibition of UGT Activities by Theaflavins in Human Recombinant UGTs

**Figure 2:**
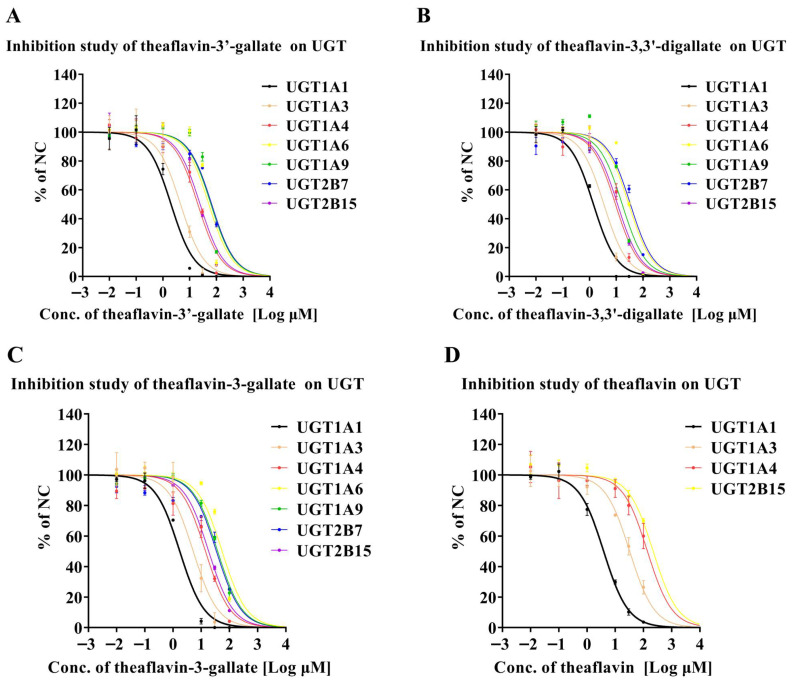
Inhibition of UGT enzymes by theaflavin derivatives in pooled HLMs. (**A**) Theaflavin-3′-gallate inhibits seven UGT enzymes: UGT1A1, UGT1A3, UGT1A4, UGT1A6, UGT1A9, UGT2B7, and UGT2B15. (**B**) Theaflavin-3,3′-digallate inhibits seven UGT enzymes: UGT1A1, UGT1A3, UGT1A4, UGT1A6, UGT1A9, UGT2B7, and UGT2B15. (**C**) Theaflavin-3-gallate inhibits seven UGT enzymes: UGT1A1, UGT1A3, UGT1A4, UGT1A6, UGT1A9, UGT2B7, and UGT2B15. (**D**) Theaflavin suppresses the functions of four UGT enzymes: UGT1A1, UGT1A3, UGT1A4, and UGT2B15. Results are expressed as mean values ± standard deviation (n = 3).

Figure 2A summarizes the direct inhibitory effect of theaflavin-3′-gallate on human recombinant UGT enzymes. Theaflavin-3′-gallate moderately inhibited UGT1A1-catalyzed beta-estradiol and UGT1A3-mediated beta-estradiol metabolism, with IC50 values of 2.02 and 4.58 μM, respectively. In contrast, it exhibited negligible inhibition on UGT1A4-catalyzed trifluoperazine, UGT1A6-mediated 4-trifluoromethyl-7-hydroxycoumarin, UGT1A9-catalyzed 4-trifluoromethyl-7-hydroxycoumarin, UGT2B7-mediated 4-trifluoromethyl-7-hydroxycoumarin, and UGT2B15-catalyzed 4-trifluoromethyl-7-hydroxycoumarin, with IC50 values exceeding 20 μM. Figure 2B illustrates the inhibitory effects of theaflavin-3,3′-digallate on human recombinant UGT enzymes. Theaflavin-3,3′-digallate showed moderate inhibition of UGT1A1-catalyzed beta-estradiol, UGT1A3-mediated beta-estradiol, and UGT1A4-catalyzed trifluoperazine, with IC50 values of 1.40, 3.44, and 9.33 μM, respectively. However, it displayed weak inhibitory effects on UGT1A9-catalyzed 4-trifluoromethyl-7-hydroxycoumarin and UGT2B15-catalyzed 4-trifluoromethyl-7-hydroxycoumarin, with IC50 values ranging from 10 to 20 μM. Theaflavin-3,3′-digallate showed minimal inhibition towards UGT1A6-mediated 4-trifluoromethyl-7-hydroxycoumarin and UGT2B7-mediated 4-trifluoromethyl-7-hydroxycoumarin, with IC50 values exceeding 20 μM. Figure 2C summarizes the inhibition of UGT activities by theaflavin-3-gallate. Theaflavin-3-gallate exhibited moderate inhibition of UGT1A1-catalyzed beta-estradiol metabolism, with an IC50 value of 1.74 μM. It also demonstrated moderate inhibition of UGT1A3-mediated beta-estradiol metabolism, with an IC50 value of 5.22 μM. However, for other UGT enzymes, such as UGT1A9-catalyzed 4-trifluoromethyl-7-hydroxycoumarin and UGT2B15-catalyzed 4-trifluoromethyl-7-hydroxycoumarin, the IC50 values ranged from 10 to 20 μM, indicating weak inhibitory effects. Additionally, theaflavin-3-gallate showed negligible inhibition on UGT1A4-catalyzed trifluoperazine, UGT1A6-mediated 4-trifluoromethyl-7-hydroxycoumarin, and UGT2B7-mediated 4-trifluoromethyl-7-hydroxycoumarin, with IC50 values exceeding 20 μM. Figure 2D demonstrates the inhibitory effect of theaflavin on UGT enzymes. Theaflavin moderately inhibited UGT1A1-catalyzed beta-estradiol metabolism, with an IC50 value of 3.89 μM. However, it exhibited negligible inhibition towards UGT1A3-mediated beta-estradiol, UGT1A4-catalyzed trifluoperazine, and UGT2B15-catalyzed 4-trifluoromethyl-7-hydroxycoumarin, with IC50 values exceeding 20 μM. Statistical analyses were performed to determine the significance of these differences. Our data indicates that different theaflavin glycoside structures exhibit significant differences in their inhibitory activities on UGT enzymes.

### 3.3. Molecular Docking of Theaflavins with Five Key Enzymes

Molecular docking simulations were performed on five key enzymes: UGT1A1, UGT1A3, UGT1A4, CYP1A2, and CYP2C8. These enzymes were selected due to their crucial roles in drug metabolism and potential interactions with theaflavins. Specifically, the UGTs were chosen for their involvement in glucuronidation, while the CYPs were selected for their role in oxidative metabolism of bioactive compounds.

#### 3.3.1. Theaflavins with UGT1A1 and UGT1A3

**Figure 3:**
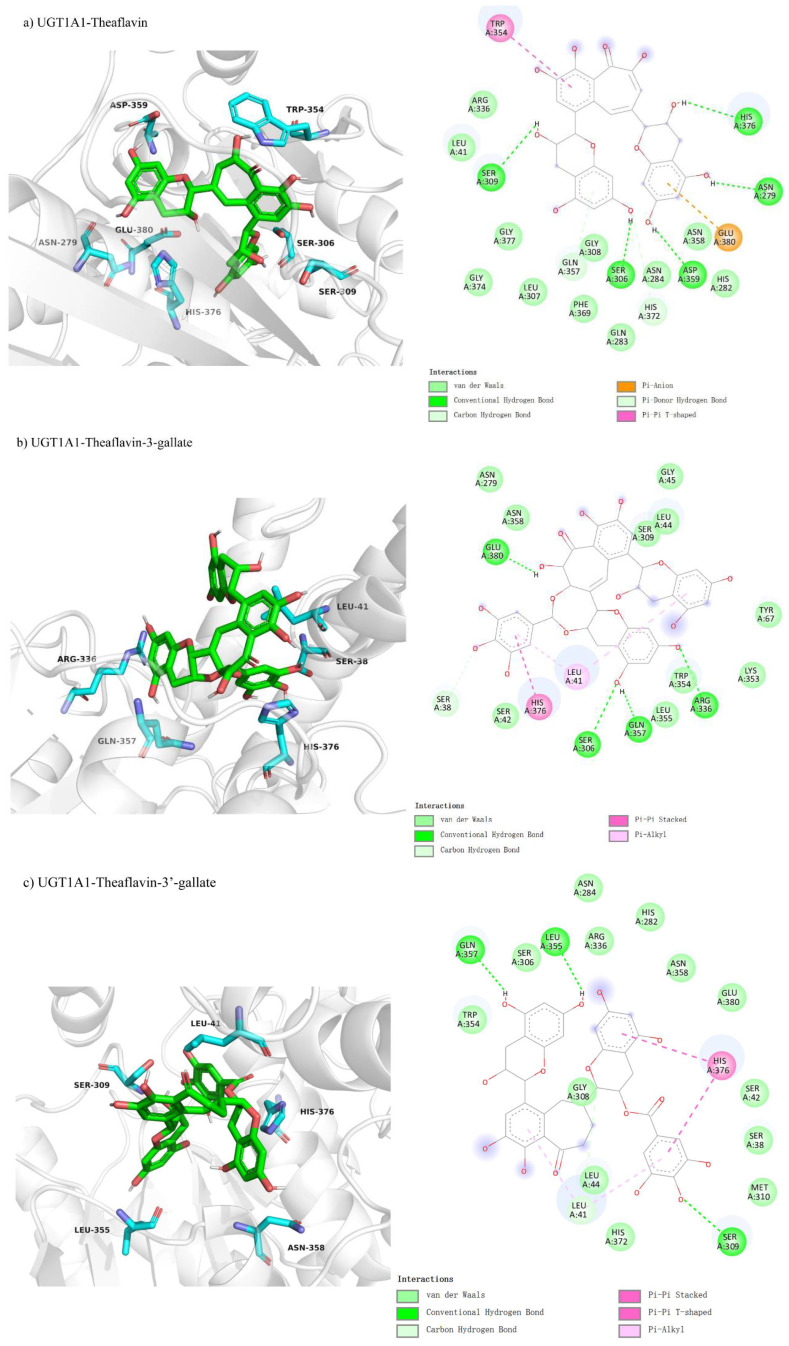

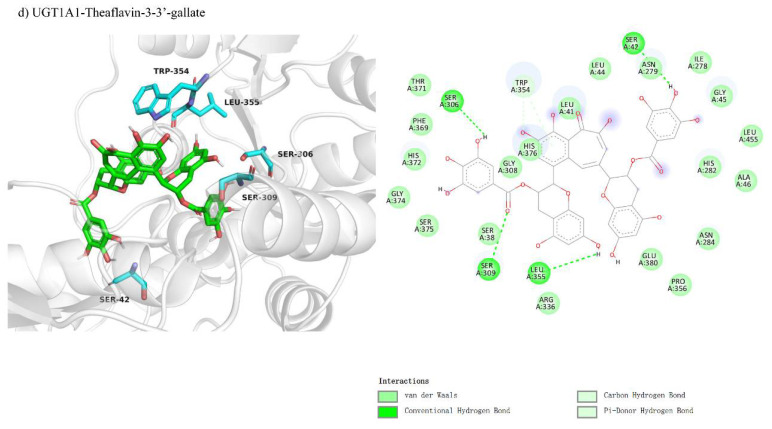
Comparison of molecular docking of UGT1A1 with theaflavin and its isomers under competitive inhibition conditions: (**a**) UGT1A1-theaflavin interaction; (**b**) UGT1A1-theaflavin-3-gallate interaction; (**c**) UGT1A1-theaflavin-3′-gallate interaction; (**d**) UGT1A1-theaflavin-3,3′-gallate interaction.

We studied theaflavin’s binding interactions with UGT1A1 using molecular docking, hypothesizing competitive inhibition. This was based on theaflavin’s structural similarity to the enzyme’s natural substrates, which could potentially disrupt substrate binding at the active site. We investigated the competitive inhibition docking interactions of UGT1A1 with four theaflavin derivatives, presented in Figure 3a–d. The docking conformation for UGT1A1 with theaflavin (Figure 3a) involves 20 residues, with 5 residues forming hydrogen bonds, resulting in a binding free energy of −8.1 kcal/mol (Table 3). In the comparison between UGT1A1 with theaflavin-3-gallate (Figure 3b) and UGT1A1 with theaflavin-3′-gallate (Figure 3c), it was found that 17 residues interact with the ligand in both cases. However, the former forms five hydrogen bond pairs, while the latter has four hydrogen bond pairs and two π-related interactions. This results in a binding free energy of −8.3 kcal/mol for the former and −8.7 kcal/mol for the latter. UGT1A1 with theaflavin-3,3′-gallate (Figure 3d) interacts with 25 residues, forming seven hydrogen bond pairs, resulting in a binding free energy of −10.2 kcal/mol. These results suggest that the presence of extra gallate groups improves the binding affinity of UGT1A1 to theaflavin derivatives, with stronger interactions attributed to increased hydrogen bonding and π-interactions. This trend is reflected in the progressively more negative binding free energy values.

**Figure 4:**
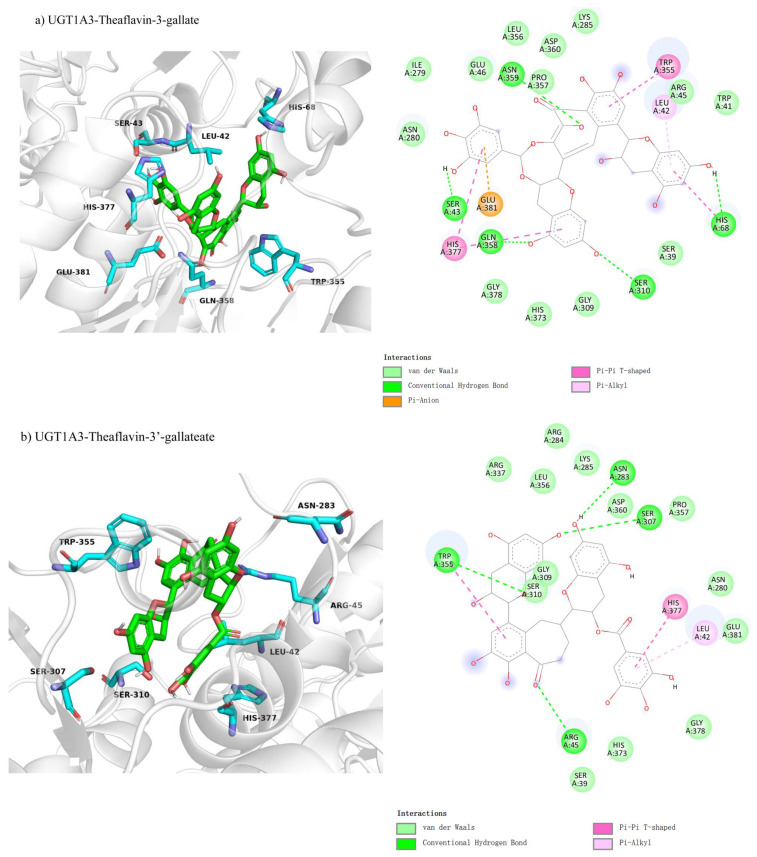

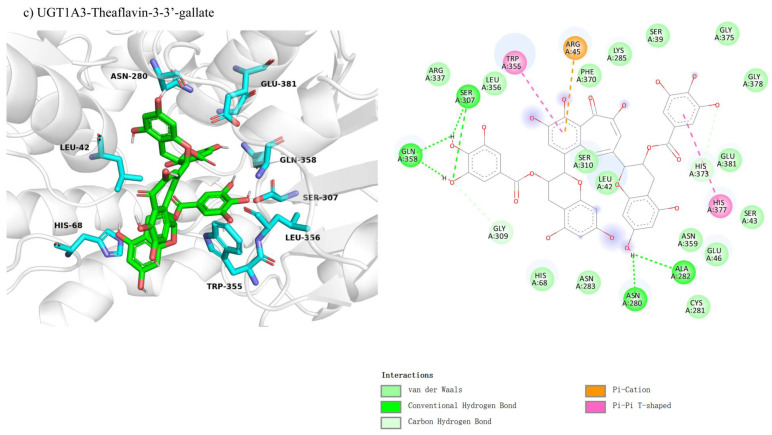
Comparison of molecular docking of UGT1A3 with theaflavin and its isomers under competitive inhibition conditions: (**a**) UGT1A3-theaflavin-3-gallate interaction; (**b**) UGT1A3-theaflavin-3′-gallate interaction; (**c**) UGT1A3-theaflavin-3,3′-gallate interaction.

We examined the competitive inhibition docking interactions of UGT1A3 with three theaflavin derivatives, shown in Figure 4a–c. The docking conformation for UGT1A3 with theaflavin-3-gallate (Figure 4a) involves 22 residues, with 5 residues forming five hydrogen bond pairs and 5 residues participating in π-related interactions, resulting in a binding free energy of −9.3 kcal/mol (Table 3). For UGT1A3 with theaflavin-3′-gallate (Figure 4b), 19 residues interact with the ligand, forming four hydrogen bond pairs and three π-related interactions, yielding a binding free energy of −8.6 kcal/mol. The docking for UGT1A3 with theaflavin-3,3′-gallate (Figure 4c) involves 26 residues, with 6 residues forming eight hydrogen bond pairs and 3 residues in π-related interactions, resulting in a binding free energy of −10.0 kcal/mol. These results indicate that theaflavin derivatives exhibit stronger binding affinities to UGT1A3 with increasing gallate group substitutions, particularly theaflavin-3,3′-gallate, which may serve as the most potent inhibitor.

#### 3.3.2. Comparison of Molecular Docking of UGT1A4, CYP1A2, and CYP2C8 with Theaflavin and Its Isomers Under Competitive Inhibition Conditions

**Figure 5:**
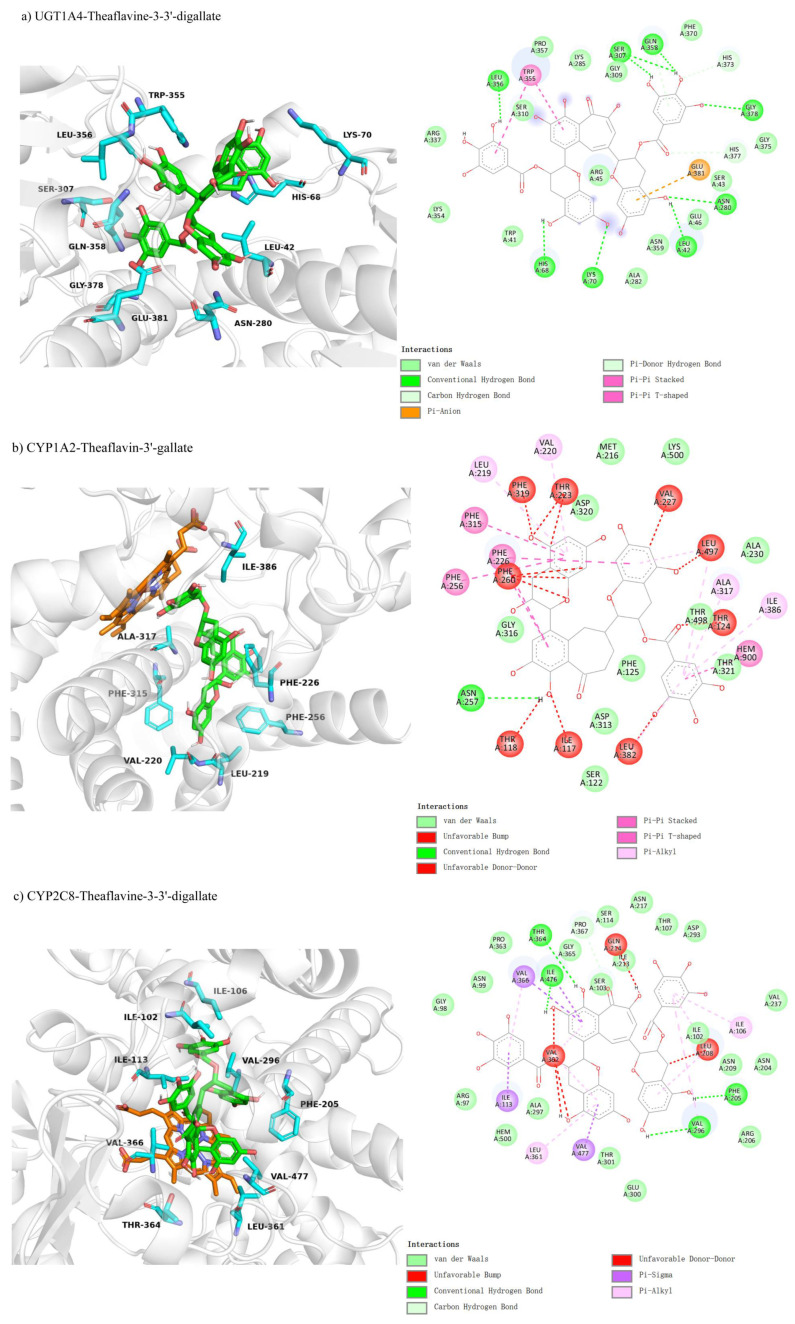
Comparison of molecular docking of UGT1A4, CYP1A2, and CYP2C8 with theaflavin and its isomers under competitive inhibition conditions: (**a**) UGT1A4-theaflavin-3,3′-gallate interaction; (**b**) CYP1A2-theaflavin-3′-gallate interaction; (**c**) CYP2C8-theaflavin-3,3′-digallate interaction.

We compared the molecular docking of UGT1A4, CYP1A2, and CYP2C8 with theaflavin and its isomers, hypothesizing competitive inhibition due to the structural similarity between theaflavin and the natural substrates of these enzymes, which may interfere with substrate binding at the active sites. The results of the docking studies are summarized as follows: The docking conformation of UGT1A4 with theaflavin-3,3′-gallate under competitive inhibition is shown in Figure 5a. In this conformation, 26 residues interact with the ligand, with 10 residues forming 12 hydrogen bond pairs, and 2 residues involved in π-related interactions, resulting in a binding free energy of −10.2 kcal/mol (Table 3). For CYP1A2 with theaflavin-3′-gallate (Figure 5b), the binding free energy was calculated to be 20.8 kcal/mol. Structural issues arose due to spatial constraints, where the docking pocket was too small to accommodate the large ligand without steric clashes. This suggests that the inhibition does not follow a competitive inhibition model, as indicated by the unrealistic forces generated (Table 3). The docking results for CYP2C8 with theaflavin-3,3′-digallate (Figure 5c) also revealed similar issues, with a binding free energy of 0.9 kcal/mol. Multiple conformations were structurally incompatible, supporting the conclusion that the inhibition is unlikely to be competitive (Table 3). Overall, these results highlight significant differences in binding affinities and docking conformations, suggesting that UGT1A4 can effectively interact with theaflavin-3,3′-gallate, while CYP1A2 and CYP2C8 exhibit steric limitations that hinder competitive inhibition.

### 3.4. Comparison of Molecular Docking of Five Key Enzymes with Theaflavin and Its Isomers Under Non-Competitive Inhibition Conditions

#### 3.4.1. Comparison of Molecular Docking of UGT1A1 with Theaflavin and Its Isomers Under Non-Competitive Inhibition Conditions

**Figure 6:**
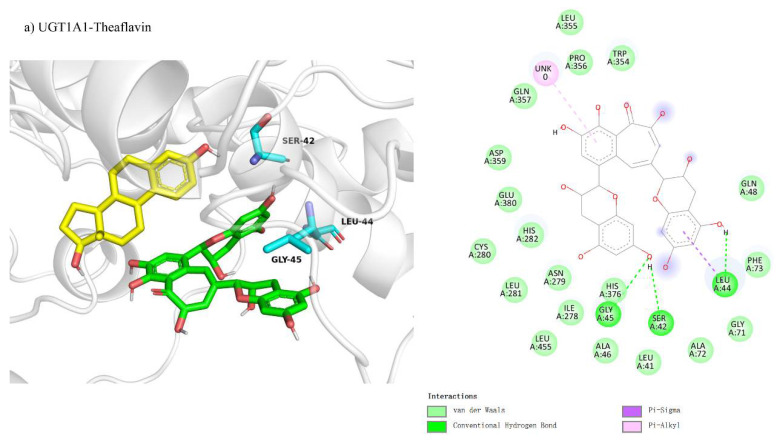

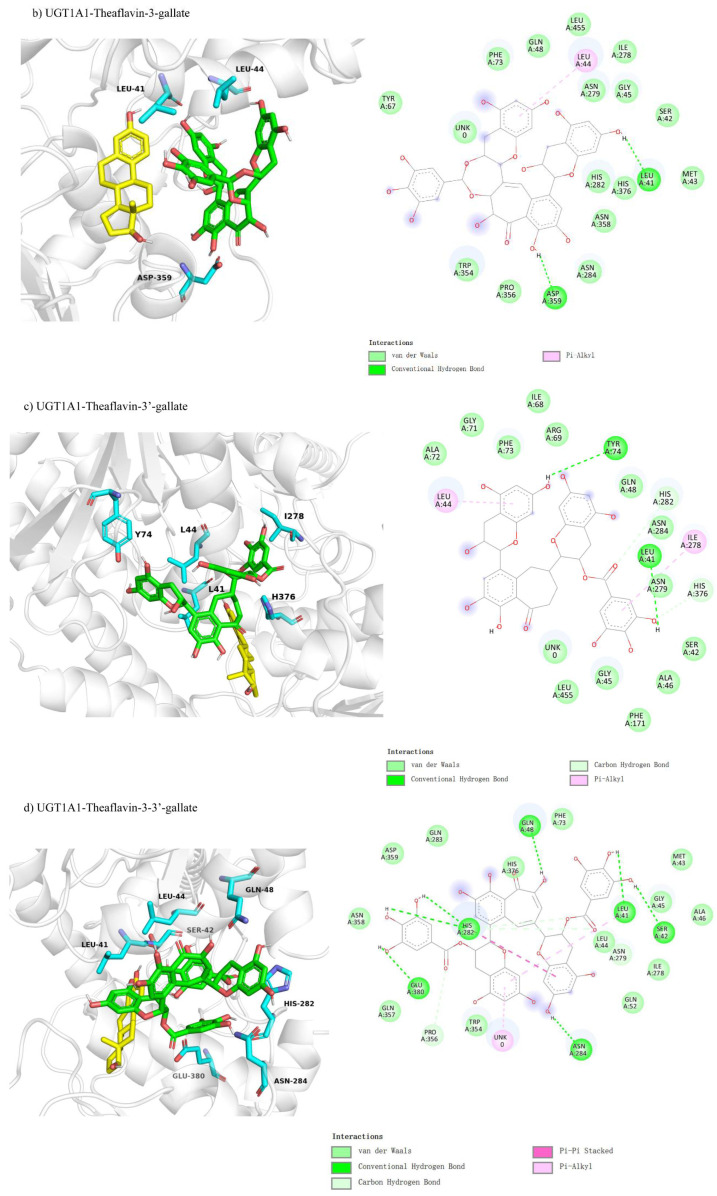
Comparison of molecular docking of UGT1A1 with theaflavin and its isomers under non-competitive inhibition conditions: (**a**) UGT1A1-theaflavin interaction; (**b**) UGT1A1-theaflavin-3-gallate interaction; (**c**) UGT1A1-theaflavin-3′-gallate interaction; (**d**) UGT1A1-theaflavin-3,3′-gallate interaction.

Molecular docking was performed to explore non-competitive inhibition interactions between UGT1A1 and theaflavin derivatives. UGT1A1-theaflavin (Figure 6a) exhibited interactions with 22 residues, forming three hydrogen bond pairs and one π-interaction, yielding a binding free energy of −9.7 kcal/mol (Table 3). The UGT1A1-theaflavin-3-gallate complex (Figure 6b) involved 18 residues, with two hydrogen bond pairs and one π-interaction, resulting in a binding free energy of −8.8 kcal/mol. For UGT1A1-theaflavin-3′-gallate (Figure 6c), 19 residues participated, forming three hydrogen bond pairs and two π-interactions, with a binding free energy of −9.0 kcal/mol. Finally, UGT1A1-theaflavin-3,3′-gallate (Figure 6d) showed interactions with 21 residues, including nine hydrogen bond pairs and two π-interactions, resulting in a binding energy of −9.6 kcal/mol. The results suggest that theaflavin-3,3′-gallate forms the strongest interactions, driven by increased hydrogen bonding and π-interactions, enhancing the overall stability of the enzyme–ligand complexes.

#### 3.4.2. Comparison of Molecular Docking of UGT1A3 with Theaflavin and Its Isomers Under Non-Competitive Inhibition Conditions

**Figure 7:**
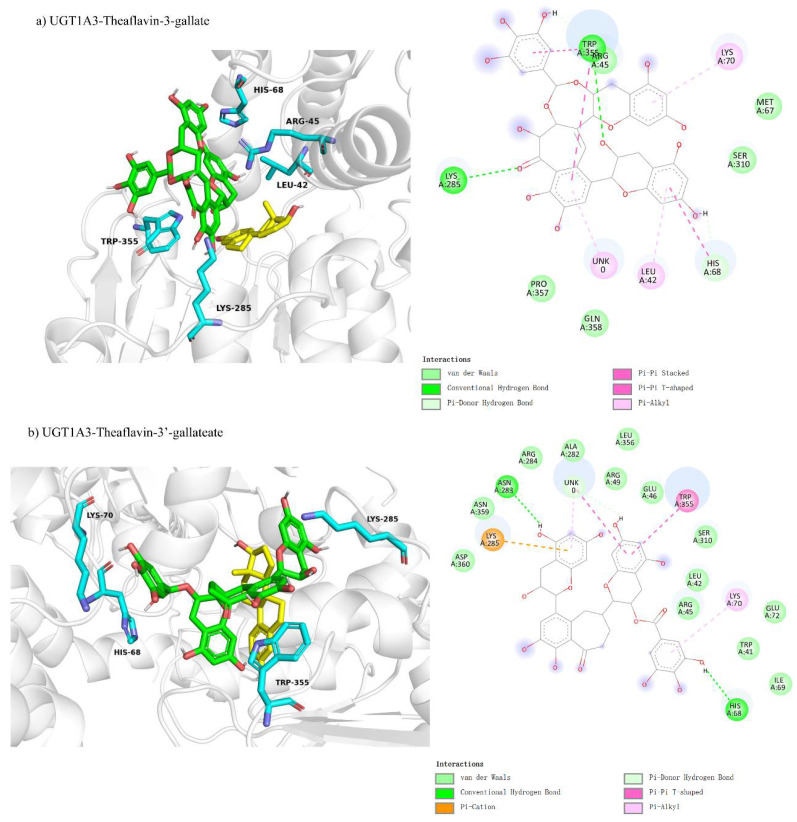

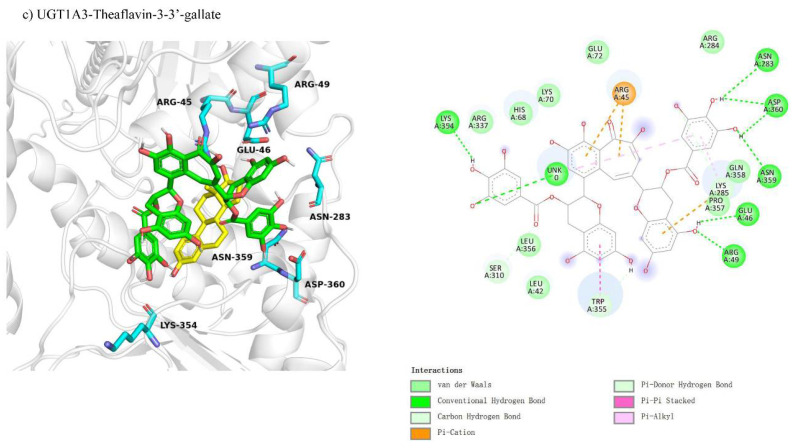
Comparison of molecular docking of UGT1A3 with theaflavin and its isomers under non-competitive inhibition conditions: (**a**) UGT1A3-theaflavin-3-gallate interaction; (**b**) UGT1A3-theaflavin-3′-gallate interaction; (**c**) UGT1A3-theaflavin-3,3′-gallate interaction.

Molecular docking was used to investigate the non-competitive inhibition interactions between UGT1A3 and various theaflavin derivatives. For UGT1A3-theaflavin-3-gallate (Figure 7a), 10 residues interacted with the ligand, forming two hydrogen bond pairs and four π-interactions, resulting in a binding free energy of −7.0 kcal/mol (Table 3). UGT1A3-theaflavin-3′-gallate (Figure 7b) involved 18 residues with two hydrogen bond pairs, three π-interactions, and additional interactions with the original substrate, resulting in a binding energy of −7.7 kcal/mol. UGT1A3-theaflavin-3,3′-gallate (Figure 7c) showed interactions with 19 residues, including nine hydrogen bond pairs and three π-interactions, leading to a binding energy of −8.3 kcal/mol. The increased binding strength with more gallate groups suggests that hydrogen bonding and π-interactions play significant roles in stabilizing the enzyme-ligand complexes.

#### 3.4.3. Comparison of Molecular Docking of UGT1A4, CYP1A2, and CYP2C8 with Theaflavin and Its Isomers Under Non-Competitive Inhibition Conditions

**Figure 8:**
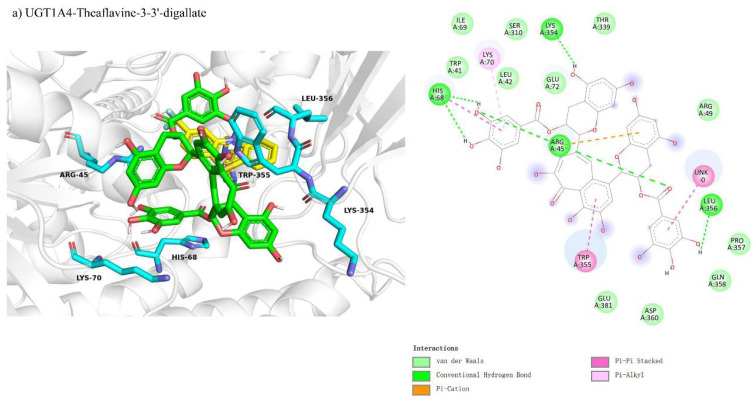

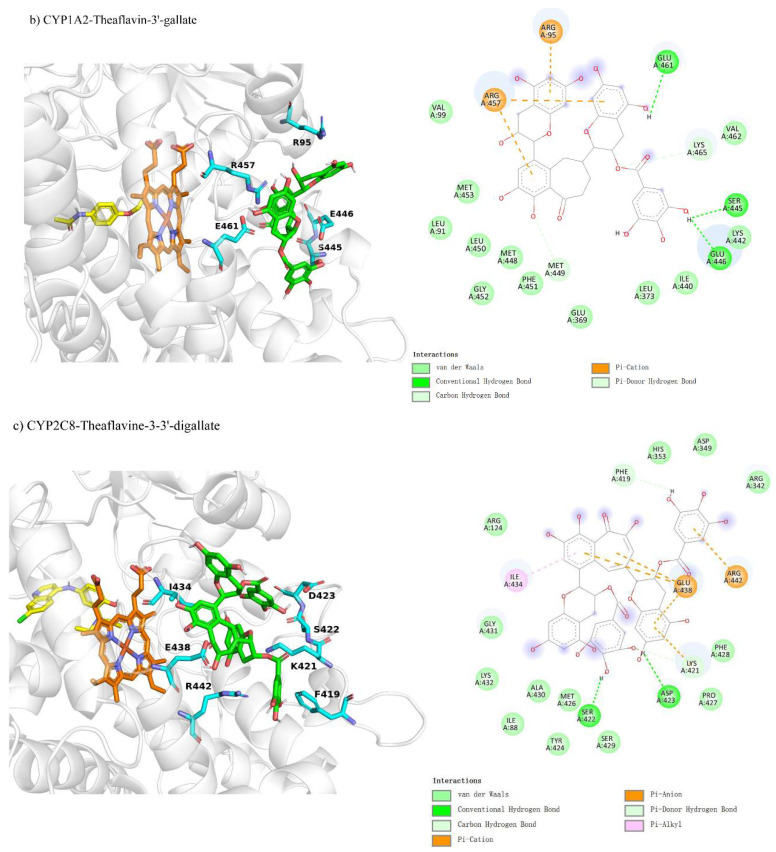
Comparison of molecular docking of UGT1A4, CYP1A2, and CYP2C8 with theaflavin and its isomers under non-competitive inhibition conditions: (**a**) UGT1A4-theaflavin-3,3′-gallate interaction; (**b**) CYP1A2-theaflavin-3′-gallate interaction; (**c**) CYP2C8-theaflavin-3,3′-digallate interaction.

Molecular docking revealed non-competitive inhibition interactions for UGT1A4 and CYP1A2 with various theaflavin derivatives. For UGT1A4-theaflavin-3,3′-digallate (Figure 8a), 17 residues interacted with the ligand, forming six hydrogen bonds and four π-interactions, with additional π-interactions involving the substrate, resulting in a binding free energy of −9.3 kcal/mol (Table 3). In CYP1A2-theaflavin-3′-gallate (Figure 8b), 19 residues participated, forming four hydrogen bonds and two π-interactions, yielding a binding free energy of −7.6 kcal/mol. This conformation likely impacts the loop behind the coenzyme, indirectly affecting the substrate catalytic pocket. For CYP1A2-theaflavin-3′-gallate (Figure 8c), 20 residues were involved, producing four hydrogen bonds and four π-interactions, with a binding free energy of −7.5 kcal/mol. This interaction similarly appears to influence the loop region rather than the catalytic pocket, suggesting an inhibitory effect through indirect modulation of the catalytic site.

## 4. Discussion

Theaflavins are key functional components with significant health benefits, including the prevention of cardiovascular diseases, diabetes, and cancer. This study focuses on four theaflavin monomers: theaflavin-3′-gallate, theaflavin-3,3′-digallate, theaflavin-3-gallate, and theaflavin. The CYP450 and UGT families are crucial enzyme systems involved in the metabolism of a wide range of endogenous and exogenous compounds. Understanding the detailed effect of theaflavins on multiple isozymes of CYP450 and UGT is essential for providing insights into their mechanisms, nutritional implications, and metabolic significance. The results showed that theaflavins exhibit moderate, weak, and even negligible inhibitory effects on isozymes of CYP450 and UGT. Studies have shown that catechins, such as (-)-epigallocatechin-3-gallate (EGCG), exert a range of inhibitory effects on UGT enzymes [21,22]. For instance, EGCG has been reported to moderately inhibit UGT1A1 and UGT1A9, similar to the moderate inhibition observed for theaflavin-3-gallate on UGT1A1 and UGT1A3 in our study. However, the inhibition spectrum of EGCG tends to be broader, affecting more UGT enzymes at lower concentrations compared to theaflavin-3-gallate. Polyphenols such as resveratrol and quercetin have also been studied for their inhibitory effects on UGT enzymes [23]. Resveratrol has been found to inhibit UGT1A1 and UGT1A6 with moderate potency, while quercetin shows strong inhibition of UGT1A1. Theaflavin-3-gallate’s moderate inhibition of UGT1A1 aligns with these findings, suggesting a common property among different polyphenols in modulating UGT enzyme activity. Compared to other polyphenols, theaflavin-3-gallate exhibits a more selective inhibition profile, with significant effects primarily on UGT1A1 and UGT1A3 [24]. This selective inhibition is less pronounced than that of some other tea polyphenols like EGCG, which affects a broader range of UGT enzymes. Theaflavins from black tea exhibit selective inhibitory effects on specific UGT enzymes, notably UGT1A1, UGT1A3, and UGT1A4. These interactions underscore the potential for significant diet–nutrient interactions, which must be considered in nutritional practice. Further research is necessary to explore the in vivo relevance of these findings and to develop guidelines for the safe consumption of theaflavin-rich products alongside various dietary components.

Our study has demonstrated the inhibitory effects of theaflavin derivatives on specific CYP450 isozymes. Specifically, theaflavin-3′-gallate was shown to moderately inhibit CYP1A2-catalyzed phenacetin metabolism, while theaflavin-3,3′-digallate exhibited a moderate inhibitory effect on CYP2C8-mediated amodiaquine metabolism. These findings contribute to a detailed understanding of the interactions between natural compounds and metabolic enzymes. The moderate inhibition observed for both CYP1A2 and CYP2C8 by theaflavin derivatives suggests that these compounds can potentially alter the metabolic profiles of nutrients and other substances metabolized by these enzymes. This is particularly relevant for dietary components with narrow safety margins or those extensively metabolized by these pathways, indicating a need for careful consideration and potential adjustments in dietary recommendations when consuming theaflavin-rich substances. The specificity of theaflavin-3′-gallate and theaflavin-3,3′-digallate towards CYP1A2 and CYP2C8, respectively, highlights a unique interaction profile that could be leveraged in the development of dietary supplements or functional foods aimed at modulating nutrient metabolism [25]. This interaction could provide a protective effect by modulating the metabolism of substances that produce potentially harmful metabolites [26]. In comparison with previous studies, our findings provide a detailed examination of specific theaflavin derivatives rather than a general assessment of tea polyphenols. Previous research has established the inhibitory potential of various tea polyphenols on CYP450 enzymes. Our study offers a more focused analysis on theaflavin-3′-gallate and theaflavin-3,3′-digallate, thereby enhancing the understanding of their distinct biochemical interactions [27,28]. Unlike studies that primarily focus on green tea catechins, this study shifts the focus to black tea theaflavins, expanding the scope of natural inhibitors of CYP450 isozymes [29]. This distinction is crucial, as it underscores the diverse metabolic effects of different tea components and their potential implications for dietary recommendations and nutrient interactions.

Our study has revealed the inhibitory effects of various theaflavin derivatives on UGT1A isoforms involved in the metabolism of beta-estradiol and trifluoperazine [30]. These findings provide critical insights into the interactions between theaflavins and metabolic enzymes, expanding our understanding of the potential impact of dietary polyphenols on nutrient metabolism. The moderate inhibition of UGT1A1 and UGT1A3 by theaflavin-3′-gallate, as well as the broader inhibitory effects of theaflavin-3,3′-digallate on UGT1A1, UGT1A3, and UGT1A4, suggest that these compounds can significantly influence the metabolism of substrates processed by these enzymes. This is particularly important for dietary components like beta-estradiol and trifluoperazine, where changes in metabolic rates can alter their nutritional efficacy and safety profiles [31]. The results underscore the need for caution when combining theaflavin-rich foods or supplements with substances metabolized by UGT1A isoforms. Our findings align with and extend these studies by providing a comparative analysis of black tea theaflavins, highlighting the distinct effects of different tea components on nutrient metabolism [4]. Unlike general polyphenol studies [32], our research offers a focused examination of the inhibitory effects of specific theaflavin derivatives. This distinction is essential, as it highlights the diverse effects of different tea components on nutrient metabolism, contributing to more tailored dietary recommendations and potential beneficial uses of theaflavins [33]. The results of our study underscore the importance of integrating dietary polyphenols into nutrient metabolism research [34]. The diverse inhibitory profiles of theaflavin derivatives on UGT1A isoforms demonstrate the complexity of diet–nutrient interactions and the necessity for personalized dietary advice, especially for individuals consuming foods metabolized by these enzymes [35,36]. Moreover, our findings are in line with and expand upon previous research on the effects of green tea catechins on UGT activity, providing a comparative analysis with black tea theaflavins. This distinction is essential, as it highlights the diverse effects of different tea components on nutrient metabolism, contributing to more tailored dietary recommendations and potential beneficial uses of theaflavins.

To contextualize the inhibitory effects of theaflavin derivatives, it is crucial to consider the physiological levels of these compounds expected in individuals consuming tea. Following the consumption of black tea, theaflavins are absorbed into the bloodstream, albeit at relatively low concentrations due to their limited bioavailability. Studies have shown that the maximum plasma concentration (C_max) of theaflavins after the ingestion of 700 mL of black tea is typically in the range of 0.1 to 1 μM [37]. Given these concentrations, the inhibitory effects observed in our in vitro experiments, where theaflavin derivatives were tested at concentrations up to 100 μM, may not directly translate to significant in vivo inhibition under normal dietary conditions. However, it is important to consider scenarios of higher intake through concentrated supplements or extracts, which could lead to higher systemic levels and potentially more pronounced interactions with UGT isoforms. Our findings suggest that while typical tea consumption is unlikely to result in theaflavin concentrations high enough to cause substantial inhibition of UGT1A isoforms, individuals consuming high doses of theaflavin-rich supplements should be aware of potential interactions. Personalized dietary advice, especially for individuals on medications metabolized by UGT1A enzymes, could mitigate any adverse effects arising from such interactions.

This study evaluated the inhibitory effects of theaflavin monomers on several CYP and UGT enzymes through molecular docking, classifying the inhibition patterns into three categories [18,38]: (1) A significant difference between competitive and non-competitive inhibition, allowing clear identification of the inhibition type; (2) a moderate difference (greater than 10%) between competitive and non-competitive inhibition, with docking results favoring the type with higher binding energy; (3) a minimal difference (less than 10%) between the two inhibition types, suggesting mixed-type inhibition. Based on our classification, several key conclusions were drawn. CYP1A2-theaflavin-3′-gallate and CYP2C8-theaflavin-3,3′-digallate demonstrated clear non-competitive inhibition. UGT1A1-theaflavin interactions indicated a tendency towards non-competitive inhibition. In contrast, UGT1A3-theaflavin-3-gallate, UGT1A3-theaflavin-3′-gallate, and UGT1A3-theaflavin-3,3′-digallate primarily exhibited competitive inhibition. For other enzyme-theaflavin interactions, molecular docking calculations could not distinguish a dominant inhibition type, implying mixed-type inhibition. These findings highlight the complexity of theaflavin interactions with hepatic enzymes and suggest that binding energy differences can influence inhibition type. However, for cases where competitive and non-competitive inhibition are indistinguishable, further experimental studies are needed to confirm the molecular docking predictions. This study provides insight into the inhibitory mechanisms of theaflavins, potentially guiding future research on enzyme modulation and nutrient metabolism.

## 5. Conclusions

The study investigated the effects of various theaflavin derivatives on CYPs and UGTs enzymes, which are critical in the metabolism of nutrients and bioactive compounds. The results demonstrated that theaflavin-3′-gallate moderately inhibited CYP1A2-catalyzed phenacetin metabolism with an IC_50_ value of 8.67 μM, and CYP2C8-mediated amodiaquine metabolism was moderately inhibited by theaflavin-3,3′-digallate, with an IC_50_ value of 6.40 μM. Both theaflavin-3′-gallate and theaflavin-3-gallate exhibited weak inhibitory effects on CYP2C8-mediated amodiaquine metabolism, CYP3A4-T-catalyzed testosterone metabolism, and CYP3A4-M-mediated midazolam metabolism, with IC_50_ values ranging from 10 to 20 μM. Theaflavin-3′-gallate showed negligible inhibition towards several other CYP-mediated reactions, including CYP2B6, CYP2C9, CYP2C19, and CYP2D6, with IC_50_ values exceeding 20 μM. Similarly, theaflavin-3-gallate exhibited negligible inhibition towards these CYP enzymes, as did theaflavin itself for a broad range of CYP-catalyzed reactions. Regarding UGT enzymes, theaflavin-3′-gallate moderately inhibited UGT1A1-catalyzed beta-estradiol and UGT1A3-mediated beta-estradiol metabolism, with IC_50_ values of 2.02 μM and 4.58 μM, respectively. Theaflavin-3,3′-digallate similarly showed moderate inhibition of UGT1A1, UGT1A3, and UGT1A4, with IC_50_ values of 1.40, 3.44, and 9.33 μM, respectively. Theaflavin-3-gallate exhibited weak inhibitory effects on UGT1A4 and UGT2B15, with IC_50_ values ranging from 10 to 20 μM. Theaflavin derivatives generally demonstrated negligible inhibition towards UGT1A6, UGT1A9, UGT2B7, and UGT2B15, with IC_50_ values exceeding 20 μM. Our study shows that theaflavin-3′-gallate and theaflavin-3,3′-digallate moderately influence the metabolic activity of CYP1A2, CYP2C8, UGT1A1, UGT1A3, and UGT1A4. These findings enhance our understanding of theaflavins as modulators of nutrient and bioactive compounds, emphasizing the potential impact of dietary factors on metabolic pathways. Theaflavin interactions with CYP1A2 and CYP2C8 suggest non-competitive inhibition, whereas interactions with UGT1A3 predominantly indicate competitive inhibition.

## Figures and Tables

**Table 1:** The probe substrates, inhibitors, and metabolites of each UGT isoform.

UGT Isoform	Probe Substrates (μM)	Inhibitor (μM)	Metabolites
UGT1A1	beta-estradiol (10)	Silibinin (100)	Estradiol-3-glucuronide
UGT1A3	beta-estradiol (10)	Quindine (300)	Estradiol-3-glucuronide
UGT1A4	Trifluoperazine (20)	Hecogenin (30)	Trifluoperazine-glucuronide
UGT1A6	4-trifluoromethyl-7-hydroxycoumarin (50)	Diclofenac (300)	4-tri-fluoromethyl-7-hydroxycoumarin glucuronide
UGT1A9	4-trifluoromethyl-7-hydroxycoumarin (10)	Diclofenac (500)	4-tri-fluoromethyl-7-hydroxycoumarin glucuronide
UGT2B7	4-trifluoromethyl-7-hydroxycoumarin (50)	Diclofenac (300)	4-tri-fluoromethyl-7-hydroxycoumarin glucuronide
UGT2B15	4-trifluoromethyl-7-hydroxycoumarin (50)	Midostaurin (50)	4-tri-fluoromethyl-7-hydroxycoumarin glucuronide

**Table 2:** The probe substrates, inhibitors, and metabolites of CYP450.

CYP450 Enzyme	Probe Substrates (μM)	Inhibitor (μM)	Metabolites
CYP1A2	Phenacetin (30)	α- Naphthoflavone (30)	Acetaminophen
CYP2B6	Bupropion (100)	Thiotepa (50)	Hydroxybupropion
CYP2C8	Amodiaquine (1.5)	Montelukast (30)	N-Desethyl Amodiaquine
CYP2C9	Diclofenac (25)	Sulfaphenazole (10)	4′-Hydroxydiclofenac
CYP2C19	S-Mephenytoin (50)	Ticlopidine (20)	S-4′-Hydroxymephenytoin
CYP2D6	Dextromethorphan (8)	Quinidine (1)	Dextrorphan
CYP3A4	Testosterone (100), Midazolam (4)	Ketoconazole (5)	6β-hydroxytestosterone, Midazolam 1-hydroxylation

**Table 3:** Comparison of binding free energies of five key enzymes (UGT1A1, UGT1A3, UGT1A4, CYP1A2, and CYP2C8) with theaflavin and its isomers under competitive and non-competitive inhibition conditions.

Enzymes	Theaflavin	Binding Free Energy at Docking Conformation (kcal/mol)	
		Competitive Inhibition	Non-Competitive Inhibition
UGT1A1	Theaflavin	−8.1	−9.7
	Theaflavin-3-gallate	−8.3	−8.8
	Theaflavin-3′-gallate	−8.7	−9.0
	Theaflavin-3-3′-gallate	−10.2	−9.6
UGT1A3	Theaflavin-3-gallate	−9.3	−7.0
	Theaflavin-3′-gallate	−8.6	−7.7
	Theaflavin-3-3′-gallate	−10.0	−8.3
UGT1A4	Theaflavin-3-3′-gallate	−10.2	−9.3
CYP1A2	Theaflavin-3′-gallate	20.8	−7.6
CYP2C8	Theaflavin-3-3′-gallate	0.9	−7.5

## Data Availability

The original contributions presented in the study are included in the article/Supplementary Materials; further inquiries can be directed to the corresponding authors.

## Supplementary Materials

The following supporting information can be downloaded at: https://www.mdpi.com/article/10.3390/foods14162822/s1, Figure S1: Theaflavin and its gallate; Table S1: Protein Data for Molecular Docking.

## Abbreviations

CYP450: Cytochrome P450 enzymes; UGTs: UDP-glucuronosyltransferases; NADPH: Nicotinamide adenine dinucleotide phosphate; UDPGA: uridine diphosphate glucuronic acid; LC-MS/MS: Liquid Chromatography-Tandem Mass Spectrometry; MRM: Multiple Reaction Monitoring; AUC: area under the curve; CID: Compound Identifier; DDI: Drug-Drug Interaction; NMPA: National Medical Products Administration; USFDA: US Food and Drug Administration; IC50: Inhibitory concentration 50%.
