# Supplementary material for: In Vitro Inhibitory Effects and Molecular Mechanism of Four Theaflavins on Isozymes of CYP450 and UGTs
Source: Foods. 2025 Aug 14;14(16):2822. doi: 10.3390/foods14162822 (PMC12385234; doi:10.3390/foods14162822)
Supplement: Supplementary file 1 [file foods-14-02822-s001.zip › foods-3755900-supplementary.pdf]

A

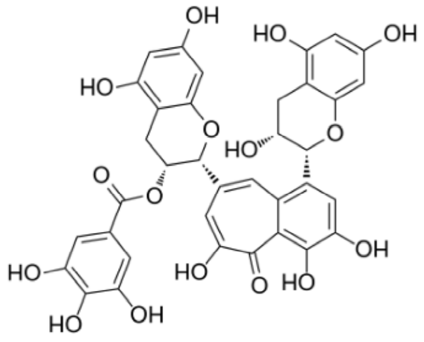

Theaflavin-3'-gallate

B

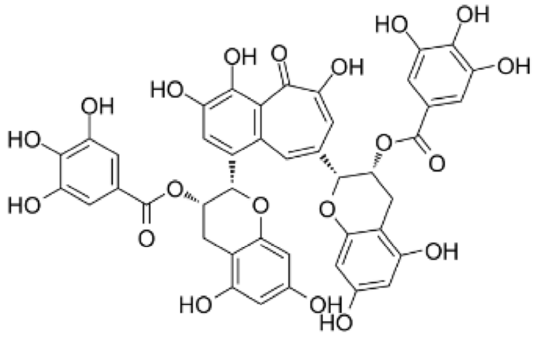

Theaflavin-3,3'-digallate

C

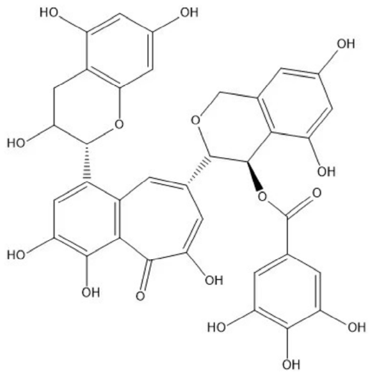

Theaflavin-3-gallate

D

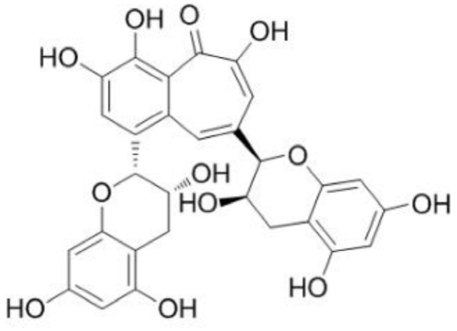

Theaflavin

Supplementary Figure S1 Theaflavin and its gallate

Supplementary Table S1: Protein Data for Molecular Docking

Refcode	Protein Name	Origin	Co-crystallized Ligand	Resolution (Å)
2HI4	CYP1A2	RCSB	α-Naphthoflavone	1.95
3IBD	CYP2B6	RCSB	Bifonazole	2.0
2NNI	CYP2C8	RCSB	Palmitoleic acid	2.8
1OG2	CYP2C9	RCSB	Warfarin	2.0
4GQS	CYP2C19	RCSB	4-(4-Chlorophenyl)imidazole	2.87
2F9Q	CYP2D6	RCSB	Prinomastat	3.0
1W0E	CYP3A4-T	RCSB	Ketoconazole	2.0
1TQN	CYP3A4-M	RCSB	Metyrapone	2.75

4WJ5	UGT1A1	RCSB	UDP-Glucuronic Acid	2.55
6NRS	UGT1A3	RCSB	UDP-Glucuronic Acid	2.8
2I3L	UGT1A4	RCSB	UDP-Glucuronic Acid	2.6
2O8H	UGT1A6	RCSB	UDP-Glucuronic Acid	2.5
4KAV	UGT1A9	RCSB	UDP-Glucuronic Acid	2.7
4FM5	UGT2B7	RCSB	UDP-Glucuronic Acid	2.6
2O6L	UGT2B15	RCSB	UDP-Glucuronic Acid	2.8
